# Experimental iodine-125 seed irradiation of intracerebral brain tumors in nude mice

**DOI:** 10.1186/1748-717X-2-38

**Published:** 2007-09-26

**Authors:** Joost JC Verhoeff, Lukas JA Stalpers, Annet W Coumou, Kees Koedooder, Cristina Lavini, Cornelis JF Van Noorden, Jaap Haveman, William P Vandertop, Wouter R van Furth

**Affiliations:** 1 Department of Radiation Oncology, Academic Medical Center, University of Amsterdam, 1105 AZ Amsterdam, The Netherlands; 2 Department of Neurosurgery, Academic Medical Center, University of Amsterdam, 1105 AZ Amsterdam, The Netherlands; 3 Department of Radiology, Academic Medical Center, University of Amsterdam, 1105 AZ Amsterdam, The Netherlands; 4 Department of Cell Biology and Histology, Academic Medical Center, University of Amsterdam, 1105 AZ Amsterdam, The Netherlands

## Abstract

**Background:**

High-dose radiotherapy is standard treatment for patients with brain cancer. However, in preclinical research external beam radiotherapy is limited to heterotopic murine models– high-dose radiotherapy to the murine head is fatal due to radiation toxicity. Therefore, we developed a stereotactic brachytherapy mouse model for high-dose focal irradiation of experimental intracerebral (orthotopic) brain tumors.

**Methods:**

Twenty-one nude mice received a hollow guide-screw implanted in the skull. After three weeks, 5 × 10^5 ^U251-NG2 human glioblastoma cells were injected. Five days later, a 2 mCi iodine-125 brachytherapy seed was inserted through the guide-screw in 11 randomly selected mice; 10 mice received a sham seed. Mice were euthanized when severe neurological or physical symptoms occurred. The cumulative irradiation dose 5 mm below the active iodine-125 seeds was 23.0 Gy after 13 weeks (BED_tumor _= 30.6 Gy).

**Results:**

In the sham group, 9/10 animals (90%) showed signs of lethal tumor progression within 6 weeks. In the experimental group, 2/11 mice (18%) died of tumor progression within 13 weeks. Acute side effects in terms of weight loss or neurological symptoms were not observed in the irradiated animals.

**Conclusion:**

The intracerebral implantation of an iodine-125 brachytherapy seed through a stereotactic guide-screw in the skull of mice with implanted brain tumors resulted in a significantly prolonged survival, caused by high-dose irradiation of the brain tumor that is biologically comparable to high-dose fractionated radiotherapy– without fatal irradiation toxicity. This is an excellent mouse model for testing orthotopic brain tumor therapies in combination with radiation therapy.

## Background

Postoperative radiotherapy is standard treatment for most patients with malignant brain tumors, including gliomas, malignant meningiomas, brain metastases, and medulloblastomas [1]. High doses of radiotherapy, i.e. 50–80 Gy or more given during 5–8 weeks, are required to control tumor growth. Despite these aggressive radiotherapy regimens, the five-year survival of patients with a brain tumor remains poor, ranging from a few percent for brain metastases, 10% for glioblastoma multiforme [2], 50% for low-grade glioma [3], 70% for childhood medulloblastoma [4], to 88% for meningioma [5]. Brachytherapy with radioactive iodine-125 (^125^I) seeds, which is effective against brain tumors, is used mostly for re-irradiation of recurrent brain tumors [6-9].

Although radiotherapy has been proven to be effective in prolonging survival and improving the quality-of-life of brain tumor patients, further research is warranted to increase its effectiveness. In glioma patients, success is expected of combination therapy using radiotherapy and either cytostatic drugs [10-12], molecular pathway modifiers [13], or hyperthermia [14,15], although none of these strategies has resulted in major improvements in prognosis.

Experimental treatment requires pre-clinical animal models. Experimental radiotherapeutic studies are usually performed with xenotransplantation of tumors in the hind leg of immunocompromised mice (heterotopic implantation) instead of implantation in the brain (orthotopic implantation) because high-dose external beam radiotherapy in the latter case is fatal for small animals [16-18]. The minimal reliable field size for external irradiation is approximately 1 cm^2 ^with the use of appropriate shielding. This is feasible for low dose treatment of e.g. rat brains, which are much larger than murine brains [19]. Due to the dimensions of smaller rodents such as mice, it is not possible to deliver a high external beam radiation dose to tumors in the brain without irradiating surrounding tissues such as the normal brain and esophagus.

In the present study, we have investigated whether fatal irradiation toxicity can be prevented by implantation of an ^125^I brachytherapy seed to enable local high-dose irradiation of an orthotopic xenotransplanted glioblastoma in the mouse brain.

## Methods

### Implantation of a hollow guide-screw in the mouse skull (day -26)

**Figure 1:**
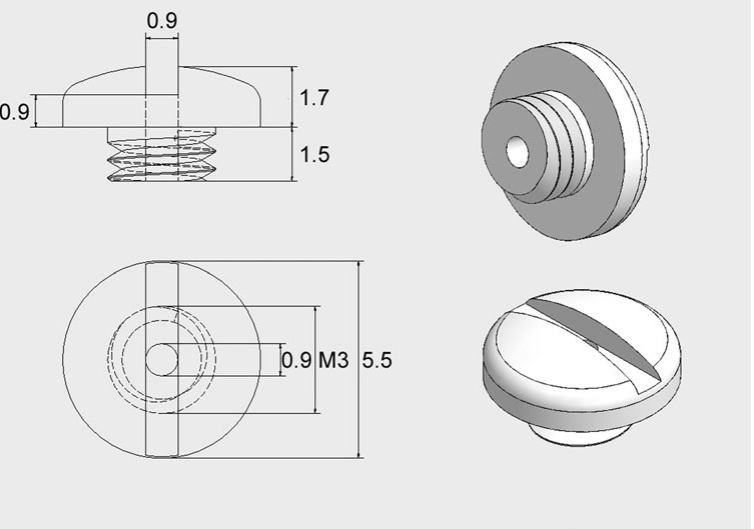
**Stereotactic screw**. The in-house developed hollow plastic stereotactic screw for implantation in the mouse skull. The inner diameter was 0.9 mm to tightly fit an ^125^I brachytherapy seed. Dimensions are given in mm. M3: metric screw thread 3.

Athymic Nude-*nu *female mice (Harlan, Horst, The Netherlands), 4–6 weeks of age, were kept in four groups of six. Prior to cancer cell injection on day -26 (day 0 is the start of therapy) a hollow guide-screw was implanted in the skull of the mouse [20]. Before surgery, each mouse was anesthetized by injection of FFM-mix (fentanyl citrate, fluanisone, midazolam, 0.1 ml per 10 gram body weight). The mice were then placed in a holder to fix the head. The skin was disinfected with iodine (1% iodine, 70% alcohol). A small incision was made and a drop of 3% lidocaine was put on the skull surface. A sterilized hand drill (9905 Dremel™, Breda, The Netherlands) was used to drill a burr-hole through the skull, in the right frontolateral position, to hold the screw (Figure 1). The skin was closed with three stitches. One hundred microliters phosphate-buffered saline were injected intraperitoneally for optimal recovery by preventing dehydration.

### Culture of glioblastoma cells

The glioblastoma cell line U251-NG2, a transfected human glioblastoma cell line with an overexpression of NG2 (provided by dr. Chekenya and prof. Bjerkvig, University of Bergen, Norway), was used for orthotopic xenotransplantation in the nude mouse brain. We selected the U251-NG2 cell line to investigate combination therapy consisting of radiotherapy and antiangiogenic drugs in future experiments. The glial precursor proteoglycan, neuron glia 2 (NG2), promotes angiogenesis-dependent tumor growth in the brain [21,22].

The cells were grown in Dulbecco's Modified Eagle's Medium (DMEM) with the addition of 10% heat inactivated newborn calf serum, four times the prescribed amount of amino acids, 800 μg/ml neomycin and 100 IU/ml penicillin/streptomycin (Gibco, Paisley, UK) [22]. Neomycin was added to select for neomycin-resistant clones that overexpress NG2. Cells were grown in culture flasks (Costar, Corning, NY, USA) in a humidified incubator at 37°C, in air mixed with 10% CO_2_. Two hours before cell transplantation, cells were harvested from the flasks by adding 2 ml trypsin. After washing, suspensions of 5 × 10^7 ^cancer cells in 300 μl PBS were prepared and kept on ice.

### Administration of U251-NG2 cells (day -5)

**Figure 2:**
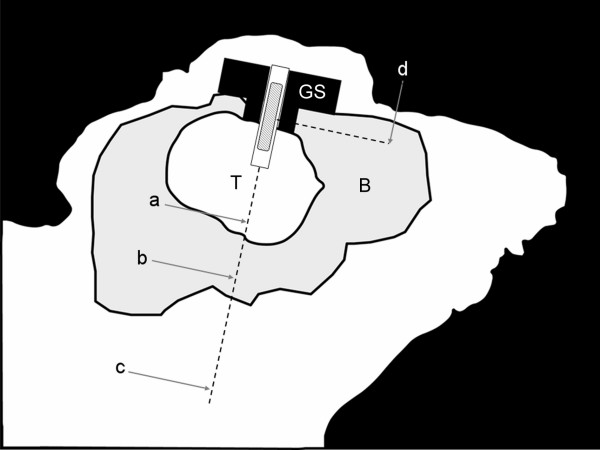
**Sagittal view**. Schematic sagittal view through the head of an intracerebral brain tumor-bearing mouse with implanted stereotactic screw containing an ^125^I brachytherapy seed. Cumulative radiation dose during 13 weeks for a 2 mCi ^125^I brachytherapy seed at four distances from the seed: (a) 2.5 mm below ('tumor dose' 52.1 Gy [integrated biological effective dose (BED) 91.2 Gy]), (b) 5 mm below ('minimal tumor dose' 23.0 Gy [integrated BED 30.6 Gy]), (c) 10 mm below ('pharynx dose' 7.8 Gy [integrated BED 8.7 Gy]) and (d) 5 mm central lateral (138.3 Gy [integrated BED 414.2 Gy]). T, tumor; B, brain; GS, subcutaneous plastic guide-screw through the skull.

Three weeks after implantation of the screw (day -5), each mouse was anesthetized with 0.07 ml FFM-mix per 10 gram body weight intraperitoneally, and fixed in the holder. The skin was disinfected, the previous incision scar was reopened, and the guide-screw was exposed. Through the central canal a microsyringe (pt.style #3; Hamilton, Bonaduz, Switzerland) was inserted. A blocker or 'arret' was attached to the syringe to be able to inject cancer cell suspensions precisely 2 mm below the screw in the right frontolateral brain (Figure 2). A suspension of 5 × 10^5 ^cancer cells in 3 μl PBS was injected slowly during 1 minute. The syringe was removed and the skin closed; the mouse recovered after injection of 0.1 ml PBS intraperitoneally.

### Iodine-125 brachytherapy seed implant (day 0)

**Figure 3:**
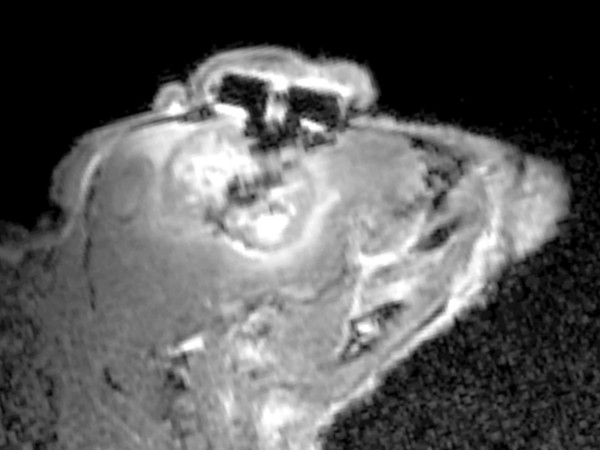
**Mouse MRI**. Magnetic resonance image (1.5 Tesla) of the head of a U251-NG2 glioblastoma-bearing mouse (sagittal view) with gadolinium contrast enhancement 61 days after cancer cell injection. No seed was inserted in this case.

Five days after cancer cell injection, each mouse was anesthetized again, the skin reopened and the guide-screw exposed. A 2 mCi ^125^I seed (model 6711; Medi-Physics, Amersham, Arlington Heights, IL, USA) or an inactive sham seed was inserted through the hole in the screw (Figures 2 and 3). Eleven mice received an experimental seed; ten mice received a sham seed; three mice died before seed implantation.

### Follow-up and euthanasia (day 1–91)

All mice were kept in a specific pathogen-free environment with optimal feeding, temperature and hygienic conditions and ample space, according to stringent experimental animal facility regulations. Mice were observed and weighed daily. Euthanasia was performed in case of extreme weight loss (more than 20 percent of body weight at the start of the experiment), the development of a domehead, occurrence of rotational behavior or other severe neurological signs, or when there were signs of dehydration. Euthanasia was performed on all surviving animals after 13 weeks by introducing CO_2 _into a sealed cage. After death, the skull was opened and the brains removed for histological analysis. All mouse experiments were approved, monitored and reviewed by the university committee for animal experiments.

After hematoxylin-eosin staining, histological analysis was performed on every twentieth 8 μm section across the entire formalin-fixed brain. Magnetic resonance imaging with gadolinium enhancement was performed in a 1.5 T MRI scanner (Signa LX, General Electric Co, Waukesha, USA). Two tumor-bearing mice without implanted seeds were scanned 61 days after cancer cell injection. Mouse survival was calculated by the Kaplan & Meier method and compared by the log rank-test in SPSS^©^.

## Results

### Animal survival

**Figure 4:**
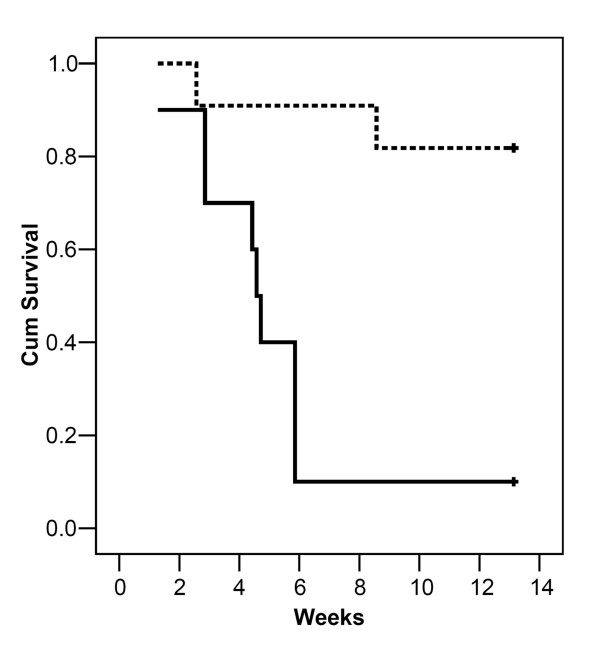
**Survival curves**. Survival curves for U251-NG2 glioblastoma-bearing mice treated with a stereotactically implanted ^125^I brachytherapy seed (intermitted line) or a sham seed (uninterrupted line).

Figure 4 shows the survival curves for 11 glioblastoma-bearing mice implanted with a radioactive ^125^I seed and 10 glioblastoma-bearing mice with a sham seed. In the sham seed group, 9/10 (90%) died from tumor progression within 6 weeks. Only 2/11 (18%) mice with the ^125^I seed died from tumor progression within 13 weeks of seed implantation. The median survival of mice implanted with sham seeds was 4.6 weeks versus more than 13 weeks for the mice implanted with radioactive seeds. The difference between the groups was highly significant (p < 0.0007). Similar results were observed in a second experiment (data not shown).

### Histological analysis

**Figure 5:**
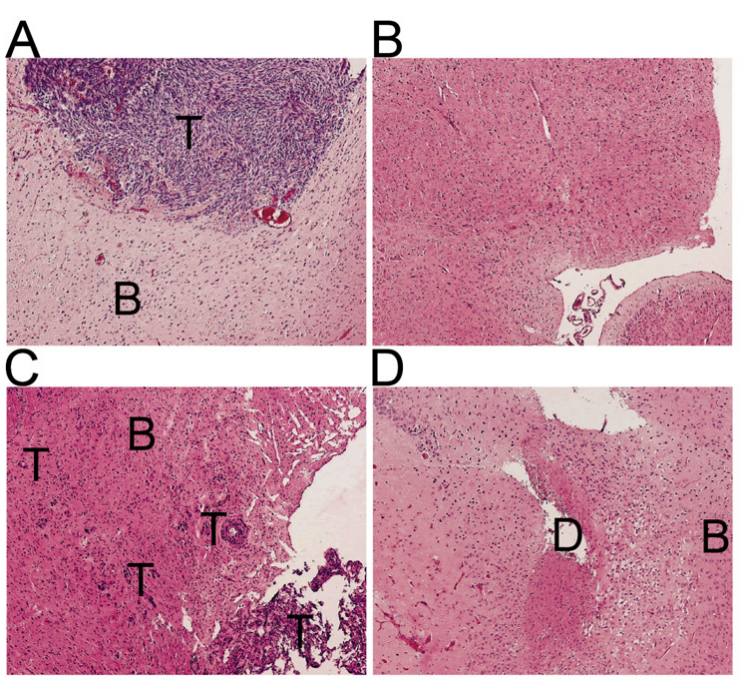
**HE-stained sections**. Hematoxylin-eosin stained sections of mouse brain. (a) Section of an non-irradiated mouse brain with a large vital glioblastoma (T) that died 4.6 weeks after sham seed implantation. Normal brain tissue (B). (b) Section of the brain of the only long-surviving mouse in the group that was implanted with a sham seed. The mouse, euthanized 91 days after implantation, showed no vital tumor. (c) Section of the brain of an irradiated mouse that died from tumor progression 18 days after implantation of the ^125^I brachytherapy seed. Diffuse infiltration of cells (T) is present in the brain. (d) Section of the brain of an irradiated mouse euthanized at 13 weeks after implantation of a ^125^I brachytherapy seed showing regions of mechanical trauma (D), hypovascular brain tissue next to normal brain (B), but no vital tumor.

In the sham-treated mice, all 9 mice that died within 6 weeks showed intracranial glioblastoma (Figure 5a). The only long-surviving sham seed-implanted mouse, which was euthanized after 13 weeks, did not show signs of a tumor (Figure 5b). Of the mice implanted with radioactive seeds, both mice that were sacrificed before the end of the study (after 2.6 and 8.6 weeks) showed vital tumor tissue (Figure 5c). The nine long-surviving irradiated mice showed either histological evidence of a few vital glioblastoma cells (n = 3; 33%) or no histological evidence of vital tumor tissue but of brain trauma or a hypovascular brain (Figure 5d).

## Discussion

Our present study describes for the first time a mouse model for high-dose radiotherapy of orthotopic brain tumors. Irradiation of tumors was performed using a guide-screw with a 2 mCi ^125^I brachytherapy seed. Irradiation led to a significantly improved survival and higher tumor control probability in U251-NG2 glioblastoma-bearing nude mice. This mouse model may serve as an experimental animal model to study the effects of combination therapy consisting of local high-dose irradiation and chemical compounds that may have an additional therapeutic effect, such as new radio-sensitizing and anti-angiogenic compounds.

The major advantage of this brachytherapy model compared to external beam (orthovoltage) irradiation is that a high radiation dose can be delivered to the murine orthotopic brain tumor without causing fatal toxicity to the normal brain and pharynx, as observed when comparable doses of external beam irradiation are delivered. Apart from a lower applicable radiation dose, external beam irradiation is more labor-intensive because of the multiple fractions of anesthetized treatment and is only applicable to much larger rodents such as rats [19].

A few points of consideration about the model include the steep dose and dose-rate-gradient around the ^125^I seed, the relatively fast ^125^I radioactive decay (half-life of 59.4 days) and the subsequent radiobiological uncertainties. However, similar or worse dosimetric problems are encountered when very small external photon beam fields are used.

Dosimetric studies have shown that the measured dose rates and the anisotropically 'apple' shaped dose rate distribution of a single seed vary within the measurement error from the seed specifications provided by the manufacturer [23]. The steep dose-rate gradient around the seed implanted in or adjacent to the tumor provides a high dose rate and cumulative tumor dose close to the seed, and thus to the tumor but a relatively low dose rate and cumulative dose farther away from the seed and thus in normal brain tissue. For example, for the 2 mCi ^125^I brachytherapy seed the cumulative radiation dose is 138 Gy after 13 weeks at 5 mm central lateral. At 5 mm below the seed, where we defined the 'minimal tumor dose', the initial dose rate is 1.7 cGy/hr and the cumulative dose is 23.0 Gy after 13 weeks.

The BED of permanent seed implants is a point of ongoing discussion [24]. The main concern is the uncertainty about the biological significance of the residual source activity after 1.5 source decay half-times of ^125^I (half-time = 59.4 days) [25]. For both ethical and evaluation purposes, we terminated our study at 13 weeks (91 days) after implant of a 2 mCi ^125^I source. After this relatively short period, we consider the BED approximations as proposed by Dale and Jones [24] to be safe for low-dose rate permanent implants removed after 90 days (see appendix for calculation details). These calculations yield a BED_tumor _2.5 mm distal of the seed of 91.2 Gy, equivalent to a conventional external beam radiotherapy dose of 76 Gy in 200 cGy daily fractions, which is more than clinically feasible.

Although dose-rate effects are of major importance for dose rates of 40 cGy/hr and higher, which is the range of clinical dose rates, this is probably of less importance for the permanent ^125^I implant (1–5 cGy/hr) [24]. During the 91 days of implantation of the 2 mCi source, the dose rate 5 mm below the source decays from 1.7 cGy/hr to 0.6 cGy/hr.

It is not clear whether these biological models can be extrapolated to even lower dose rates after 2–3 decay half-times for seeds with a lower initial activity, such as the 0.5 mCi sources that we use clinically for prostate cancer implants [26], or in tumors with a very high doubling time. This is not necessarily a disadvantage of our mouse brachytherapy model; on the contrary, the model allows in vivo study of these uncertainties of clinical brachytherapy.

## Conclusion

Guide-screw implanted ^125^I brachytherapy seeds can be used for experimental clinically significant irradiation of an orthotopic glioblastoma implanted in the nude mouse brain. The method is feasible, gives a high tumor control probability and an improved survival rate of the U251-NG2 glioblastoma-bearing mouse, without fatal irradiation toxicity.

The major concern of the model is the steep dose-rate gradient around the ^125^I seed with a correspondingly high dose inhomogeneity in the tumor. Because of the very high tumor control rate obtained in this model with little margin for any gain from additive experimental drugs, we recommend to use weaker ^125^I sources (1 mCi) for future studies.

## Competing interests

The author(s) declare that they have no competing interests.

## Appendix

Calculation of the Biological Equivalence Dose (BED) of permanent iodine-125 implants (according to Dale & Jones, 1998):

According to the linear-quadratic model the biological effective dose (BED) of fractionated external beam radiotherapy can be given as:

BED=n∗d(d+αβ)αβ
 MathType@MTEF@5@5@+=feaafiart1ev1aaatCvAUfKttLearuWrP9MDH5MBPbIqV92AaeXatLxBI9gBaebbnrfifHhDYfgasaacH8akY=wiFfYdH8Gipec8Eeeu0xXdbba9frFj0=OqFfea0dXdd9vqai=hGuQ8kuc9pgc9s8qqaq=dirpe0xb9q8qiLsFr0=vr0=vr0dc8meaabaqaciaacaGaaeqabaqabeGadaaakeaacqWGcbGqcqWGfbqrcqWGebarcqGH9aqpcqWGUbGBcqGHxiIkcqWGKbazdaWcaaqaaiabcIcaOiabdsgaKjabgUcaRmaalmaaleaaiiGacqWFXoqyaeaacqWFYoGyaaGccqGGPaqkaeaadaWcdaWcbaGae8xSdegabaGae8NSdigaaaaaaaa@3F3B@

where *n *= number of daily fractions, *d *= fraction dose (in Gray), and the *α/β *ratio is the parameter for cellular survival. It is reasonable to assume an *α/β*-ratio of 3 Gy^2 ^for normal tissue and 10 Gy^2 ^for rapidly proliferative tissue, such as tumor and squamous cell epithelium.

For a permanent implant delivering an initial dose rate R_o_, and involving a nuclide with decay constant *λ*, DNA-recovery constant *μ*, and cell-survival characteristics *α/β*, BED is given as:

BED=R0λ(1+R0(μ+λ)(αβ))
 MathType@MTEF@5@5@+=feaafiart1ev1aaatCvAUfKttLearuWrP9MDH5MBPbIqV92AaeXatLxBI9gBamXvP5wqSXMqHnxAJn0BKvguHDwzZbqegyvzYrwyUfgarqqtubsr4rNCHbGeaGqiA8vkIkVAFgIELiFeLkFeLk=iY=Hhbbf9v8qqaqFr0xc9pk0xbba9q8WqFfeaY=biLkVcLq=JHqVepeea0=as0db9vqpepesP0xe9Fve9Fve9GapdbaqaaeGacaGaaiaabeqaamqadiabaaGcbaGaemOqaiKaemyrauKaemiraqKaeyypa0ZaaSaaaeaadaqfqaqabSqaaiabicdaWaqab0qaaiabdkfasbaaaOqaaGGaciab=T7aSbaadaqadaqaaiabigdaXiabgUcaRmaalaaabaWaaubeaeqaleaacqaIWaamaeqaneaacqWGsbGuaaaakeaadaqadaqaaiab=X7aTjabgUcaRiab=T7aSbGaayjkaiaawMcaamaabmaabaWaaSWaaSqaaiab=f7aHbqaaiab=j7aIbaaaOGaayjkaiaawMcaaaaaaiaawIcacaGLPaaaaaa@55E0@

The mono-exponential recovery constant *μ *is related to the repair half-life T_1/2 _by:

μ=0.693T1/2
 MathType@MTEF@5@5@+=feaafiart1ev1aaatCvAUfKttLearuWrP9MDH5MBPbIqV92AaeXatLxBI9gBaebbnrfifHhDYfgasaacH8akY=wiFfYdH8Gipec8Eeeu0xXdbba9frFj0=OqFfea0dXdd9vqai=hGuQ8kuc9pgc9s8qqaq=dirpe0xb9q8qiLsFr0=vr0=vr0dc8meaabaqaciaacaGaaeqabaqabeGadaaakeaaiiGacqWF8oqBcqGH9aqpdaWcaaqaaiabicdaWiabc6caUiabiAda2iabiMda5iabiodaZaqaaiabdsfaunaaBaaaleaacqaIXaqmcqGGVaWlcqaIYaGmaeqaaaaaaaa@3864@

Average repair-half-lives for mammalian tissues are usually 0.5–3 h, with increasing evidence that tumor recovery half-lives are probably shorter than those for late reacting normal tissues. Assuming a tumor recovery half-life T_1/2 _= 1.5 h, than *μ *= 0.462 per hour.

The radioactive source decay constant *λ *is related to the decay half-life T_1/2 _by:

λ=0.693T1/2
 MathType@MTEF@5@5@+=feaafiart1ev1aaatCvAUfKttLearuWrP9MDH5MBPbIqV92AaeXatLxBI9gBaebbnrfifHhDYfgasaacH8akY=wiFfYdH8Gipec8Eeeu0xXdbba9frFj0=OqFfea0dXdd9vqai=hGuQ8kuc9pgc9s8qqaq=dirpe0xb9q8qiLsFr0=vr0=vr0dc8meaabaqaciaacaGaaeqabaqabeGadaaakeaaiiGacqWF7oaBcqGH9aqpdaWcaaqaaiabicdaWiabc6caUiabiAda2iabiMda5iabiodaZaqaaiabdsfaunaaBaaaleaacqaIXaqmcqGGVaWlcqaIYaGmaeqaaaaaaaa@3862@

The decay half-life T_1/2 _of iodine-125 is 59.4 days = 1425.6 hours. The decay constant is 0.000486 per hour.

For a non-permanent implant with a decaying source, removed after time T:

BED=R0λ(1−e−λT)[1+2R0λ(μ−λ)(αβ)(A−BC)]
 MathType@MTEF@5@5@+=feaafiart1ev1aaatCvAUfKttLearuWrP9MDH5MBPbIqV92AaeXatLxBI9gBaebbnrfifHhDYfgasaacH8akY=wiFfYdH8Gipec8Eeeu0xXdbba9frFj0=OqFfea0dXdd9vqai=hGuQ8kuc9pgc9s8qqaq=dirpe0xb9q8qiLsFr0=vr0=vr0dc8meaabaqaciaacaGaaeqabaqabeGadaaakeaacqWGcbGqcqWGfbqrcqWGebarcqGH9aqpdaWcaaqaaiabdkfasnaaBaaaleaacqaIWaamaeqaaaGcbaacciGae83UdWgaamaabmaabaGaeGymaeJaeyOeI0Iaemyzau2aaWbaaSqabeaacqGHsislcqWF7oaBcqWGubavaaaakiaawIcacaGLPaaadaWadaqaaiabigdaXiabgUcaRmaalaaabaGaeGOmaiJaemOuai1aaSbaaSqaaiabicdaWaqabaGccqWF7oaBaeaadaqadaqaaiab=X7aTjabgkHiTiab=T7aSbGaayjkaiaawMcaamaabmaabaWaaSWaaSqaaiab=f7aHbqaaiab=j7aIbaaaOGaayjkaiaawMcaaaaadaqadaqaamaalaaabaGaemyqaeKaeyOeI0IaemOqaieabaGaem4qameaaaGaayjkaiaawMcaaaGaay5waiaaw2faaaaa@56EC@

where:

A=12λ[1−e−2λT]
 MathType@MTEF@5@5@+=feaafiart1ev1aaatCvAUfKttLearuWrP9MDH5MBPbIqV92AaeXatLxBI9gBaebbnrfifHhDYfgasaacH8akY=wiFfYdH8Gipec8Eeeu0xXdbba9frFj0=OqFfea0dXdd9vqai=hGuQ8kuc9pgc9s8qqaq=dirpe0xb9q8qiLsFr0=vr0=vr0dc8meaabaqaciaacaGaaeqabaqabeGadaaakeaacqWGbbqqcqGH9aqpdaWcaaqaaiabigdaXaqaaiabikdaYGGaciab=T7aSbaadaWadaqaaiabigdaXiabgkHiTiabdwgaLnaaCaaaleqabaGaeyOeI0IaeGOmaiJae83UdWMaemivaqfaaaGccaGLBbGaayzxaaaaaa@3C82@

B=1(μ+λ)[1−e−(μ+λ)T]
 MathType@MTEF@5@5@+=feaafiart1ev1aaatCvAUfKttLearuWrP9MDH5MBPbIqV92AaeXatLxBI9gBaebbnrfifHhDYfgasaacH8akY=wiFfYdH8Gipec8Eeeu0xXdbba9frFj0=OqFfea0dXdd9vqai=hGuQ8kuc9pgc9s8qqaq=dirpe0xb9q8qiLsFr0=vr0=vr0dc8meaabaqaciaacaGaaeqabaqabeGadaaakeaacqWGcbGqcqGH9aqpdaWcaaqaaiabigdaXaqaamaabmaabaacciGae8hVd0Maey4kaSIae83UdWgacaGLOaGaayzkaaaaamaadmaabaGaeGymaeJaeyOeI0Iaemyzau2aaWbaaSqabeaacqGHsislcqGGOaakcqWF8oqBcqGHRaWkcqWF7oaBcqGGPaqkcqWGubavaaaakiaawUfacaGLDbaaaaa@4301@

*C *= 1 - *e*^-*λT*^

## Authors' contributions

JJCV performed the experiments, carried out the histological analysis and drafted the manuscript, LJAS participated in the design of the study, assisted with surgery and helped to draft the manuscript, AWC performed parts of the experiments, KK calculated radiation doses, CL performed mouse MRI's, CJFV participated in the design of the study and helped to draft the manuscript, JH participated in the design of the study, WPV helped to draft the manuscript, WRvF conceived of the study, and participated in its design and coordination, performed surgery and helped to draft the manuscript. All authors read and approved the final manuscript.
